# Survival, incidence, and predictors of diabetic neuropathy among type 2 diabetic patients in hospitals of Addis Ababa

**DOI:** 10.3389/fcdhc.2024.1386426

**Published:** 2024-05-02

**Authors:** Eden Tilahun, Abdata Workina, Asaminew Habtamu, Hailu Tufa, Fikadu Abebe, Ayele Fikadu, Fulea Atomsa

**Affiliations:** ^1^ National Public Emergency Contact Center, Ethiopian Public Health Institute, Addis Ababa, Ethiopia; ^2^ School of Nursing, Jimma University, Jimma, Oromia, Ethiopia; ^3^ School of Midwifery, Jimma University, Jimma, Oromia, Ethiopia; ^4^ Department of Emergency and Critical Care, Saint Paul Hospital Millennium Medical College, Addis Ababa, Ethiopia; ^5^ Independent Researcher, Beke, Oromia, Ethiopia

**Keywords:** diabetes mellitus, neuropathy, type 2 diabetes, diabetic complication, insulin

## Abstract

**Background:**

Diabetic neuropathy is a very common complication of diabetes mellitus. Thus, measuring the incidence of diabetic neuropathy is a key element in tracking the progress of epidemics of diabetes mellitus and an indication of early accessibility for healthcare in terms of type 2 diabetic patients.

**Objective:**

To assess survival, incidence, and predictors of diabetic neuropathy among type 2 diabetic patients in hospitals of Addis Ababa from June 25 to August 25, 2023.

**Methods:**

An institutional-based retrospective follow-up study design was used among newly diagnosed type 2 diabetic patients at hospitals of Addis Ababa. A chart review tool that contains socio-demographic, clinical, and comorbidity characteristics, biochemical characteristics, and the status of type 2 patients was used. A cleaned data was exported from Epi-data manager 4.6 version to SPSS version 25 for analysis. Bivariate Cox regression analysis was done to identify predictors of diabetic neuropathy at a 95% confidence level.

**Result:**

A total of 414 type 2 diabetic patients were included in the study. Of these, 97 (23.4%) developed diabetic neuropathy. Variables like having hypertension (AHR 11.25, 95% CI 3.73–33.93), anemia (AHR 4.18, 95% CI 1.78–9.82), high-density lipoprotein < 40 mg/dl (AHR 5.07, 95% CI 1.38–18.67), high creatinine level (AHR 14.67, 95% CI 4.27–50.40), diabetic retinopathy (AHR 4.32, 95% CI 1.32-14.18), and diabetic nephropathy (AHR 2.50, 95% CI 1.09–6.57) were associated with the incidence of diabetic neuropathy. The mean time to develop diabetic neuropathy was 4.94 years, CI (4.50–5.38), and the mean survival time was 6.61 years.

**Conclusion:**

The incidence of diabetic neuropathy was high relative to other studies. Variables like having hypertension, anemia, high-density lipoprotein, high creatinine level, diabetic retinopathy, and diabetic nephropathy were predictors of diabetic neuropathy. The mean time to develop diabetic neuropathy was 5 years, with a survival mean time of 7 years.

## Introduction

Diabetes mellitus is becoming increasingly prevalent in both high-income and low-income countries with its complication (1–3). It can lead to micro-vascular (nephropathy, retinopathy, and neuropathy) and macro-vascular complications (4–6). Besides to managing hyperglycemia, diabetic patients require clinical monitoring and assessment of additional risk factors, as well as managing potential predictors of complications (6–8). The incidence of diabetic neuropathy is increasing even though its existing cases is relatively reduced in sub-Saharan Africans (9).

Diabetic neuropathy (DNP) is the most common complication of diabetes mellitus (10, 11). According to a systematic review conducted in Latin America, its prevalence can range from 21.3 to 34.5% in type 2 DM and type 1 diabetes mellitus between 7.0% and 34.2% (6). Even though around half of diabetic patients are asymptomatic for DNP, most patients present with numbness, tingling, pain, and weakness that cause disability worldwide (12–15). It affects quality of life due to chronic pain, a high risk of falls, limb amputation, and foot ulceration. These manifestations of DNP further lead to sleep disorders, anxiety, and depression (6, 10, 15).

Diabetic neuropathy is a worldwide healthcare problem for both low-income and high-income countries (16, 17). It is estimated that every 30 seconds somewhere in the world, a lower limb amputation is performed due to diabetic neuropathy (18). Diabetic neuropathy is the rapidly increasing complication of diabetes mellitus imposing socio-economic burden and disability globally (7, 19–21). It accounts for 80% of foot ulceration and 50–60% of non-traumatic limb amputations (15). The pooled prevalence of diabetic neuropathy among patients with diabetes ranges from 22–46.5% worldwide (6). In Africa and Ethiopia, it ranges between 22–66% and 52.2–53.6% of diabetic patients developed diabetic neuropathy, respectively (22–24). The prevalence and incidence of diabetic neuropathy are high in developing countries due to late diagnosis, inadequacy of screening and diagnosing resources, poor control of blood glucose, a rise in the cost of health expenditures, shortage of medical resources, and lack of quality diabetic care (20, 22).

A study conducted at Black Lion Hospital showed that diabetic neuropathy is the predominant diabetic complication, which accounts for 47.5% (1). Despite studies being conducted on the prevalence of diabetic neuropathy in Ethiopia, there is no study conducted regarding the incidence of diabetic neuropathy, which is most important over prevalence to identify the rate or status of new case occurrence and measure the extent of early detection of diabetes mellitus complications. Thus, a study was aimed to determine the incidence of diabetic neuropathy and its predictors among patients with type 2 diabetes mellitus.

## Patients and methods

### Study design and setting

An institutional-based retrospective follow-up study was employed among three randomly selected public hospitals in Addis Ababa from June 25 to August 25, 2023. The three selected hospitals were Tikur Anbessa Specialized Hospital, Minilik II Hospital, and Yekatit 12 Hospital. Addis Ababa is the capital city of Ethiopia and the largest city in Ethiopia, with a population of 3,475,952 according to the 2007 population census and an annual growth rate of 2.7%. Addis Ababa city has 41 hospitals (13 public and 28 NGO and private).

### Study populations

Selected (sampled) newly diagnosed type 2 diabetes mellitus among selected public hospitals in Addis Ababa was the source population.

### Eligibility criteria

Newly diagnosed type 2 patients (those diagnosed from July 5, 2013 to July 5, 2023) aged ≥18 years old were followed for the development of an event (diabetic neuropathy) at selected hospitals were included in the study. Patients with signs and symptoms or who have already developed diabetic neuropathy and patients with an unknown date of DM diagnosis were excluded from the study.

### Sample size determination and sampling procedure

The required sample size (424) was determined via Stata software using power analysis for the log-rank test with the assumption of a 95% confidence interval, 5% margin of error, 80% power and the risk of developing diabetic neuropathy among patients with anemia is 1.68 times higher than their counterparts (AHR1.68; 95% CI: 1.03-2.76) (25).

The study participants were selected by systematic sampling technique by calculating the interval from a predetermined sample size and a total of 7,281 patients from the three selected hospitals. The required sample size for each hospital was allocated proportionally.

### Data collection tool and procedure

For the data collection, an English version checklist was developed from up-to-date similar literatures that contains socio-demographic characteristics, clinical and comorbidity characteristics, biochemical characteristics of type 2 DM patients, and outcomes of type 2 diabetes mellitus patients (5, 13, 14, 22, 25). During data collection, the diagnosis of diabetic neuropathy was considered after ruling out other potential explanations, the physicians contemplate the existence of symptoms and sign of peripheral nerve damage and abnormality of nerve conduction velocity confirmed using electromyography. Data was collected by 4BSc nurses and trained by the investigator about the content of the checklist in detail. The outcome of the follow-up was dichotomized into event (those patients diagnosed with diabetic neuropathy) and censored (patients who did not develop diabetic neuropathy or died or lost follow-up or transfer-out before developing diabetic neuropathy) within the study period. Patient chart with missing variables were excluded from the study during data collection.

### Data quality assurance

Before the data collection period, the checklist was pretested on 5% of the sample size of randomly selected newly diagnosed type two diabetic patients at Alert Hospital. The reliability coefficient for the pretest was 0.85. Training was given to data collectors on the parts of the checklist. During the period of data collection, the investigators were provided on-site close supervision and technical support, and all filled-out checklists were checked daily for completeness, accuracy, clarity, and consistency.

### Data processing and analysis

Cleaned data was entered into Epidata Manager 4.6 versions. Then, it was exported and analyzed using SPSS version 25.0. Descriptive statistics, such as percent, frequency, and mean, were used to summarize the categorical variables. Dependent variables were dichotomized into an event (developing diabetic neuropathy), and the event did not occur as a censor. The Cox regression model’s fitness was checked by graphing residual plots like the Cox-Snell residual. Bivariate Cox regression analysis was done to identify associations between dependent and independent variables. In the bivariate Cox regression, predictor variables having a P-value < 0.25 were candidates for multivariate Cox regression. The crude hazard ratio and adjusted hazard ratio, 95%CI, and P-value were used to assess the strength of the association and statistical significance. The Kaplan–Meier (KM) curve was used to compare survival time between groups of categorical variables.

### Ethical considerations

Before starting data collection, a letter of permission was obtained from the Addis Ababa Medical and Business College School of General Public Health Ethical Review Committee and given to the administrators of selected public hospitals in Addis Ababa. Permission was granted to review patients’ registration charts. The patient’s names were omitted from the checklist. The confidentiality of all the documents to be reviewed was highly secured throughout the data collection phase of the research process. Informed consent was waived by the Addis Ababa Medical and Business College School of General Public Health Ethical Review Committee due to the anonymity of the data.

## Result

### Sociodemographic, clinical, and comorbidity characteristics of the study participants

A total of 414 diabetic patients were included in the study, with a completion rate of 97.6%. Among the study participants, 228 (55.1%) of them were male, and two-thirds (70.5%) of them were aged below 60 years with a mean age of 51.2 years, which can be 11.8 years below and above the mean. Concerning the residence of the study participants, 331 (80.0%) of them were residing in the urban area (**Table 1**).

**Table 1:** Socio-demographic, clinical, and comorbidity characteristics of diabetic patients at TASH, Y12H, and Minilik II Hospital, 2023.

Variables		Frequency (n=414)	Percent
Sex	Male	228	55.1
	Female	186	44.9
Age in years	<60	292	70.5
	>=60	122	29.5
Residence	Rural	83	20.0
	Urban	331	80.0
Duration of DM in yrs.	<5	227	54.8
	≥5	187	45.2
Mean ± SD duration of DM in yrs.	4.6 ± 2,2		
Family history of diabetes mellitus	Yes	182	44.0
	No	232	56.0
Type of DM treatment	Oral	313	75.6
	Insulin	13	3.1
	Mixed	88	21.3
Family history of complications of DM	Yes	62	15.0
	No	352	85.0
Diabetic retinopathy	yes	102	24.6
	No	312	75.4
Diabetic nephropathy	Yes	126	30.4
	No	288	69.6
Cardiovascular disease	No	235	56.8
	Yes	179	43.2
Hypertension	Yes	108	26.1
	No	306	73.9
Anemia	No	252	60.9
	Yes	162	39.1
Comorbidity	Cancer	24	5.8
	Dental decay	38	9.2
	HIV	11	2.7
	Itching on the eye	12	2.9
	Ischemic stroke	26	6.3

Among the study participants, 182 (44.0%) of them had a family history of diabetes mellitus, three-fourths (313.6%) of them were treated with oral anti-hyperglycemic drugs, and 13 (3.1%) of them took insulin injections, while 88 (21.3%) of the patients took both oral and injection drugs. The mean duration of diabetes mellitus among patients were 4.6 years with standard deviation of 2.2 years. Of the study participants, one-fourth, 102 (24.6%), had diabetic retinopathy, and 126 (30.4%) had developed diabetic nephropathy. The common comorbidities the study participants had were cancer, dental decay, and HIV: 24 (5.8%), 38 (9.2%), and 11 (2.7%), respectively (**Table 1**).

### Biochemical characteristics, incidence of diabetic neuropathy, and outcomes among type 2DM patients

From the total number of study participants, 154 (37.2%) had fasting blood sugar > 125 mg/dl, and 111(26.8%) had low-density lipoprotein > 100 mg/dl, and only 157 (37.9%) of them had high-density lipoprotein > 40 mg/dl. Furthermore, 159 (38.4%) of the diabetic patients had a triglyceride level >150 mg/dl, and 152 (36.7%) of the study participants had total cholesterol > 200 mg/dl. Around one-fourth, 99 (23.9%), and one-third, 127 (32.6%), of the study participants had positive for proteinuria and albuminuria respectively. Among the study participants, 97 (23.4%) developed diabetic neuropathy. Among the study participants, one-tenth, 41 (9.9%), were deceased during the follow-up (**Table 2**).

**Table 2:** Biochemical characteristics, incidence of diabetic neuropathy and outcomes of type 2 DM patients at TASH, Y12H and Minilik II Hospital, 2023.

Variables		Frequency (n=414)	Percent
Fasting blood sugar level	> 125mg/dl	260	62.8
	≤125mg/dl	154	37.2
Low-density lipoprotein	> 100mg/dl	111	26.8
	< 100mg/dl	303	73.2
High-density lipoprotein	> 40mg/dl	157	37.9
	< 40mg/dl	257	62.1
Triglyceride level	>150mg/dl	159	38.4
	< 150mg/dl	255	61.6
Creatinine level	> 1.1mg/dl	157	37.9
	< 1.1mg/dl	257	62.1
Total cholesterol	> 200mg/d	152	36.7
	< 200mg/dl	262	63.3
Proteinuria	Positive	99	23.9
	negative	315	76.1
Albuminuria	Positive	127	32.6
	Negative	263	67.4
Diabetic neuropathy	Yes	97	23.4
	No	317	76.6
Outcomes of T2DM	Dead	41	9.9
	Alive	373	90.1

### Predictors of diabetic neuropathy among newly diagnosed T2DM patients

Based on bivariate Cox regression, predictors like age, sex, type of DM treatment, family history of DM complications, diabetic retinopathy, diabetic nephropathy, hypertension, anemia, fasting blood sugar level, creatinine level, and proteinuria were associated with the incidence of diabetic neuropathy.

Based on multivariable Cox-regression analysis, variables like having hypertension (AHR 11.25, 95% CI 3.73–33.93), being male (AHR 0.19, 95% CI 0.09–0.40), age ≥60 years (AHR 4.20, 95% CI 1.41–12.54), having anemia (AHR 4.18, 95% CI 1.78–9.82), high-density lipoprotein < 40 mg/dl (AHR 5.07, 95% CI 1.38–18.67), and creatinine level (AHR 14.67, 95% CI 4.27–50.40) were associated with the development of diabetic neuropathy. Furthermore, having diabetic retinopathy and diabetic nephropathy has increased the hazard of developing diabetic neuropathy by 4.4 (AHR 4.32 (95% CI 1.32-14.18)) and 2.5 (AHR 2.50 (95% CI 1.09-6.57)), respectively (**Table 3**).

**Table 3:** Predictors of diabetic neuropathy among newly diagnosed T2DM patients at TASH, Y12H, and Minilik II Hospital, 2023.

Covariates		Incidence of diabetic neuropathy		p-value	95%CHR (CI)	95%AHR (CI)
		Censored	Event			
		Frequency	Frequency			
Sex	Male	194	34	0.006*	0.66 (0.42-1.03)	0.19 (0.09-0.40)
	Female	123	63		1.00	1.00
Age in years	<60	211	81	0.013*	1.00	1.00
	≥60	106	16		0.62 (0.36-1.08)	4.20 (1.41-12.54)
Residence	Rural	58	25	0.916	0.98 (0.61-1.56)	
	Urban	259	72		1.00	
Family history of diabetes mellitus	No	181	51	0.354	1.22 (0.80-1.87)	
	Yes	136	46		1.00	
Type of DM treatment	Oral	240	73	0.001	0.17 (0.08-0.36)	1.43 (.61-3.35)
	Insulin	12	1		0.28 (0.04-2.29)	.07 (.01-.42)
	Mixed	64	24		1.00	1.00
Family history of complications of DM	Yes	51	11	0.127	1.68 (0.86-3.29)	3.32 (.95-11.61)
	No	266	86		1.00	1.00
Diabetic retinopathy	yes	70	32	0.007*	1.68 (0.86-3.29)	4.32 (1.32-14.18)
	No	247	65		1.00	1.00
Diabetic nephropathy	No	91	35	0.026*	1.00	1.00
	Yes	226	62		1.58 (1.01-2.46)	2.50 (1.09-6.57)
Cardiovascular disease	No	186	49	0.303	1.00	
	Yes	131	48		1.25 (0.82-1.93)	
Hypertension	No	94	14	0.014	1.00	1.00
	Yes	223	83		0.65 (0.35-1.20)	11.25 (3.73-33.93)
Anemia	No	152	50	0.005*	1.00	1.00
	Yes	165	47		0.66 (0.42-0.90)	4.18 (1.78-9.82)
Fasting blood sugar level	>125mg/dl	103	51	0.005	0.54 (0.35-0.83)	1.43 (1.07-4.35)
	≤125mg/dl	214	46		1.00	
Low-density lipoprotein	>100mg/dl	73	38	0.415	0.83 (0.52-1.31)	
	<100mg/dl	244	59		1.00	
High-density lipoprotein	> 40mg/dl	84	73	0.001*	1.00	1.00
	< 40mg/dl	233	24	3.15 (1.81-5.47)	5.07 (1.38-18.67)
Triglyceride level	>150mg/dl	121	38	0.415	0.83 (0.52-1.31)	
	<150mg/dl	196	59		1.00	
Creatinine level	>1.1mg/dl	97	60	0.012*	0.69 (0.43-0.90)	14.67 (4.27-50.40)
	≤1.1mg/dl	220	37		1.00	1.00
Total cholesterol	> 200mg/d	92	60	0.417	0.84 (0.55-1.29)	
	< 200mg/dl	225	37		1.00	
Proteinuria	Positive	86	13	0.026*	0.51 (0.28-0.92)	14.64 (4.36-49.14)
	negative	231	84		1.00	1.00
Albuminuria	positive	102	25	0.916	0.98 (0.61-1.56)	
	negative	191	72		1.00	

*p-value <0.05 in multivariate Cox regression.

CHR, crude hazard ratio; AHR, adjusted hazard ratio; CI, confidence interval.

### Time to diabetic neuropathy and survival time

The mean time to develop diabetic neuropathy was 4.94 years CI (4.50-5.38), and the median time to develop diabetic neuropathy was 5.00 years CI (4.64-5.36). Furthermore, the mean survival time following the development of diabetic neuropathy was 6.61 years, with a median survival time of 7.00 years (**Table 4**).

**Table 4:** Time to diabetic neuropathy and survival time among newly diagnosed T2D at TASH, Y12H, and Minilik II Hospital, 2023.

Means and Medians for Time to develop diabetic neuropathy			
Mean		Median	
Estimate (yrs.)	95% CI (upper-lower bound)	Estimate (yrs.)	95% CI (upper-lower bound)
4.94	4.50-5.38	5.00	4.64-5.36
Means and Medians for Survival Time			
Mean		Median	
Estimate (yrs.)	Estimate (yrs.)	Estimate (yrs.)	95% CI (upper-lower bound)
6.61	6.18-7.04	7.00	6.50-7.50

**Figure 1:**
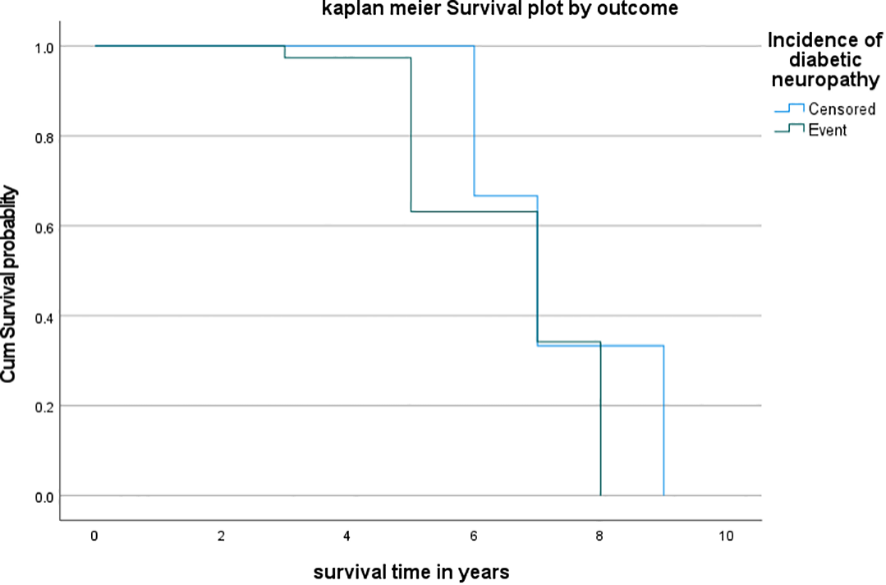
Kaplan–Meier survival estimate among patients developing diabetic neuropathy at TASH, Y12H and Minilik II Hospital, 2023.

The Kaplan-Meier survival estimate shows that the hazards of death were higher among patients developing diabetic neuropathy than their counterparts (**Figure 1**).

## Discussion

This study found that the incidence of diabetic neuropathy was 97 (23.4%). This study finding was relatively consistent with a study conducted in Vietnam, which showed that the incidence of diabetic neuropathy among people with T2DM is 26.6% (4). Furthermore, the current study finding was in line with an international study conducted between 2013 and 2015 in 14 countries and a meta-analysis study conducted in the Republic of Ireland, which showed that the overall new cases of diabetic neuropathy were 26.71% and 3.3% to 32% of the diabetic patients developed diabetic neuropathy (11, 26).

On the other hand, this study’s finding was higher than a study conducted in India and Latin America on patients with diabetes mellitus, which elucidated that the incidence of diabetic neuropathy among patients with diabetes is 12.5% and 13.7% (95% CI: 10.6–17.2), respectively (6, 9). The possible reason for this discrepancy might be due to healthcare accessibility since early treatment of diabetes mellitus can tackle its progress and complications. And also population characteristics difference might cause discrepancy among studies. However, the incidence of diabetic neuropathy in the current study was lower than other studies conducted in Germany among diabetic patients, which showed that the prevalence of diabetic neuropathy among patients with type 2 DM is 42.2% (27). And cross-sectional study conducted at Tikur Anbessa Specialized Hospital shows that 57 (47.5%) of the diabetic patients experienced diabetic neuropathy (1). This discrepancy occurred because those studies included old cases (prevalence of diabetic neuropathy). Besides, this study excluded type 1 diabetic patients which might result in potential differences in diabetic neuropathy incidence between Type 1 and Type 2 diabetes.

Besides, this study found that variables like having hypertension (AHR 11.25, 95% CI 3.73–33.93), being male (AHR 0.19, 95% CI 0.09–0.40), age ≥60 years (AHR 4.20, 95% CI 1.41–12.54), having anemia (AHR 4.18, 95% CI 1.78–9.82), high-density lipoprotein < 40 mg/dl (AHR 5.07, 95% CI 1.38–18.67), and high creatinine level (AHR 14.67, 95% CI 4.27–50.40) were associated with the development of diabetic neuropathy Furthermore, having diabetic retinopathy and diabetic nephropathy has increased the hazard of developing diabetic neuropathy by 4.4 (AHR 4.32; 95% CI 1.32-14.18) and 2.5 (AHR 2.50; 95% CI 1.09-6.57), respectively. This study finding was congruent with a study conducted in India, which revealed that nephropathy and higher creatinine levels were significantly correlated (p<0.05) with diabetic neuropathy (9). Besides, a study conducted in China was consistent with the current study, which elucidated that age > 66 years and hypertension were significant predictive factors for the development of DNP (12). Another study is also consistent with this study finding, which showed that co-morbidities such as diabetic nephropathy and retinopathy are independently associated with diabetic neuropathy (p<0.05) (27). Additionally, this study finding was consistent with a retrospective follow-up study conducted in the Debra Markos specialized hospital that elucidated that being aged > 60 years (AHR 2.93; 95% CI: 1.29–6.66), having diabetic retinopathy (AHR 2.76; 95% CI: 1.84–4.16), having anemia (AHR 3.62; 95% CI: 2.46–5.33), and having hypertension (AHR 3.22; 95% CI: 2.10–4.93) were the predictors of diabetic neuropathy (5). This showed that preventing these preventable factors can reduce the occurrence of diabetic neuropathy among type 2 diabetic patients. Additionally, our study finding also showed that, sex of participants were associated with the incidence of diabetic neuropathy (being male AHR 0.19; 95% CI 0.09-0.40). This study is supported by a study conducted in the UK which revealed that female sex is a risk factor for painful diabetic peripheral neuropathy (28). However, this study was inconsistent with other studies (29, 30). This might be due to our study included only public hospitals, which might cause patient demographics to change within different categories of hospitals.

Nevertheless, this study was contrary to a study conducted in Vietnam that showed predictors like triglyceride (OR = 1.50, 95% CI 1.11–2.03, p = 0.009) and albumin (AOR = 0.85, 95% CI 0.75–0.95, p = 0.005) were associated with DPN (4). The possible reason for this variation might be due to study design differences (retrospective study design); even early detection, time of parameter measurement; that is whether variables were measured during initial patient visit or after intervention was provide for the diagnosis and screening can make the difference, and other factors also occur due to comorbidities. Again, this study finding was inconsistent with another study conducted in Eastern Libya, which showed higher fasting blood sugar (OR = 3.51, 95% CI = 1.99–6.21) was associated with diabetic neuropathy (22). The possible justification for this variability might be to early detection and early management can affect the blood sugar level. Furthermore, our study excluded the type 1 diabetic patients that cause potential difference in predictors of diabetic neuropathy among type 1 and type diabetic patients. In the context of the Ethiopian healthcare system controlling comorbidities and early screening reduce the occurrence of diabetic neuropathy and early interventions improve the management of diabetic neuropathy.

## Limitations and strengths of the study

This study tried to generalize the incidence of diabetic neuropathy with 10 years of retrospective follow-up. However, this study did not include type 1 diabetic patients, even though the risk of diabetic neuropathy was low relative to type 2 diabetic patients. Also, due to the retrospective nature of the study design, unable-to-study factors include the educational level, income level, and lifestyle habits (smoking, physical exercise and alcohol consumption) status of the study participants.

## Conclusions

The incidence of diabetic neuropathy was high relative to other studies. Variables like having hypertension, age, having anemia, high-density lipoprotein, creatinine level, having diabetic retinopathy, and diabetic nephropathy were independent predictors of diabetic neuropathy among type 2 diabetic patients. The mean time to develop diabetic neuropathy was 5 years, with a survival mean time of 7 years.

## Data availability statement

The original contributions presented in the study are included in the article/supplementary material. Further inquiries can be directed to the corresponding author.

## Ethics statement

The studies involving humans were approved by Addis Ababa Medical and Business College School of General Public Health Ethical Review Committee. The participants provided their written informed consent to participate in this study. The animal study was approved by Addis Ababa Medical and Business College School of General Public Health Ethical Review Committee. The study was conducted in accordance with the local legislation and institutional requirements.

## Author contributions

ET: Writing – review & editing, Writing – original draft. AW: Writing – original draft, Writing – review & editing. AH: Writing – review & editing, Writing – original draft. HT: Writing – review & editing, Writing – original draft. FAb: Writing – review & editing, Writing – original draft. AF: Writing – review & editing, Writing – original draft. FAt: Writing – review & editing, Writing – original draft.
